# Evaluation of the Impact of External Conditions on Arm Positioning During Punches in MMA Fighters: A Comparative Analysis of 2D and 3D Methods

**DOI:** 10.3390/s25113270

**Published:** 2025-05-22

**Authors:** Dariusz Skalski, Magdalena Prończuk, Petr Stastny, Kinga Łosińska, Miłosz Drozd, Michal Toborek, Piotr Aschenbrenner, Adam Maszczyk

**Affiliations:** 1 Faculty of Physical Culture, Gdansk University of Physical Education and Sport, 80336 Gdansk, Poland; dariusz.skalski@answalcz.pl (D.S.); magdalena.pronczuk@awf.gda.pl (M.P.); kinga.losinska@awf.gda.pl (K.Ł.); piotr.aschenbrenner@awf.gda.pl (P.A.); 2 Department of Physiology and Biochemistry, Faculty of Physical Education and Sport, Charles University, 16000 Prague, Czech Republic; petr.stastny@ftvs.cuni.cz; 3 Institute of Sport Sciences, Jerzy Kukuczka Academy of Physical Education, 40065 Katowice, Poland; m.drozd@awf.katowice.pl; 4 Department of Biochemistry and Molecular Biology, Miller School of Medicine, University of Miami, Miami, FL 33136, USA; mtoborek@med.miami.edu

**Keywords:** motion analysis, fatigue, joint angles, punch dynamics, combat sports

## Abstract

**Highlights:**

**What are the main findings?**

- 2D analysis provides reliable estimates of joint angles (ICC = 0.81–0.99) even under fatigue and balance disruption.
- Shoulder joint angles are more consistent than elbow joint angles across all test conditions.

**What is the implication of the main finding?**

- 2D video analysis is suitable for practical biomechanical assessment in MMA training.
- Combining 2D and 3D analysis can enhance personalized coaching strategies and injury prevention.

**Abstract:**

Mixed Martial Arts (MMA) is a highly dynamic combat sport that requires precise motor coordination and technical execution. Video-based motion analysis, including two-dimensional (2D) and three-dimensional (3D) motion capture systems, plays a critical role in optimizing movement patterns, enhancing training efficiency, and reducing injury risk. However, the comparative validity of 2D and 3D systems for evaluating punching mechanics under external stressors remains unclear. This study aimed to first validate the measurement agreement between 2D and 3D motion analyses during sagittal-plane punches, and second, to examine the impact of fatigue and balance disruption on arm kinematics and punch dynamics in elite MMA athletes. Twenty-one male MMA fighters (mean age: 24.85 ± 7.24 years) performed standardized straight right punches (SRPs) and swing punches (SPs) under three experimental conditions: normal, balance-disrupted, and fatigued. Participants were instructed to deliver maximal-effort punches targeting a designated striking pad placed at a consistent height and distance. Each punch type was executed three times per condition. Kinematic data were collected using the my Dartfish Express(version 7.2.0) app (2D system) and MaxPRO infrared motion capture system (3D system). Statistical analyses included Pearson’s correlation coefficients, one-way analysis of variance (ANOVA), and linear mixed models (LMMs). Strong correlations (r = 0.964–0.999) and high intraclass correlation coefficient (ICC) values (0.81–0.99) confirmed the high reliability of 2D analysis for sagittal-plane techniques. Fatigue significantly decreased punch velocity and impact force (*p* < 0.01), while increasing joint angle variability (*p* < 0.01). These findings highlight the complementary use of 2D and 3D motion capture methods, supporting individualized monitoring, adaptive technique evaluation, and performance optimization in combat sports.

## 1. Introduction

Mixed Martial Arts (MMA) is an intense and complex combat sport that requires the integration of technical, tactical, physical, and psychological competencies [1]. MMA fighters must execute techniques with explosive power and minimal reaction times, demanding high-level neuromuscular control and kinematic precision [2]. Consequently, video-based biomechanical analysis has become an essential component of training strategies, enabling coaches to assess technical accuracy, mitigate injury risk, and enhance motor efficiency [3].

Although motion analysis technologies have been widely adopted in combat sports, few studies have evaluated their sensitivity to external stressors commonly encountered during competition. This study addresses this gap by investigating how fatigue and balance disruption affect arm positioning during straight and swing punches in elite MMA athletes. Furthermore, it assesses the comparative utility and validity of two motion analysis approaches, i.e., 2D and 3D approaches, in capturing these dynamics under varying conditions. The choice to focus specifically on straight and swing punches was deliberate: both techniques are biomechanically consistent, predominantly sagittal or sagittal-transverse in trajectory, and offer sufficient standardization for validating 2D and 3D systems under experimental conditions. More complex strikes, such as hooks or uppercuts, typically involve higher degrees of axial rotation and out-of-plane motion, which would have introduced confounding variability, particularly under fatigue or balance disruption. Including such techniques would have compromised the methodological clarity required for equivalence testing. Therefore, while this design limits the generalizability of findings to multi-planar techniques, it provides a rigorous foundation for understanding motion capture reliability in sagittal-plane-dominant strikes.

Two-dimensional (2D) and three-dimensional (3D) video analysis techniques differ markedly in terms of resolution, depth perception, operational cost, and field applicability. Two-dimensional analysis, typically implemented via smartphone cameras or applications such as my Dartfish Express, offers a low-cost, portable alternative but lacks rotational tracking and spatial depth [4]. In contrast, 3D motion capture systems such as MaxPRO deliver precise multi-planar data, accurately capturing asymmetries and complex joint trajectories, albeit at substantially higher costs and infrastructure requirements [5,6].

Recent theoretical frameworks in motor control research emphasize that movement variability serves as an adaptive mechanism rather than merely reflecting noise [7,8]. Specifically, under fatigue or external perturbations, skilled performers exhibit structured variability that enables task success despite altered physiological conditions. In combat sports, where striking dynamics involve complex coordination of multiple body segments, understanding how external stressors modulate variability is critical. Fatigue-induced changes in neuromuscular coordination have been shown to selectively alter joint kinematics and temporal sequencing [2], while balance disruptions may challenge postural control strategies essential for striking effectiveness [9]. Integrating these perspectives, the present study not only evaluates the mechanical outcomes of external conditions but also interprets upper-limb kinematic adaptations through the lens of movement variability and motor control resilience.

The biomechanical assessment of striking mechanics has long been used to evaluate performance effectiveness and injury susceptibility in combat sports. Despite the extensive literature on striking biomechanics in boxing and traditional stand-up disciplines, Mixed Martial Arts (MMA) presents unique and underexplored challenges. Unlike boxing, which involves highly constrained, rule-specific striking patterns, MMA integrates a broader range of techniques, including vertical and rotational punches executed from varying stances, dynamic transitions between striking and grappling, and performance under unpredictable, real-time constraints. This complexity introduces greater biomechanical variability and demands more versatile neuromuscular control. Thus, MMA provides a more ecologically valid context for evaluating how external conditions such as fatigue and balance disruption affect striking mechanics. Furthermore, the scientific literature on punch kinematics in MMA remains limited compared to boxing, highlighting a critical gap that this study aims to address. Research in boxing and kickboxing has emphasized that optimal punch mechanics depend on kinetic chain coordination, segmental alignment, and balance control [7]. Elite-level athletes typically exhibit shorter punch durations and higher impact forces, attributed to superior motor timing and optimized joint angles [8]. Similar patterns have been observed in kickboxing, where minor deviations in hip and shoulder rotation significantly reduce punch force and precision [9].

The role of fatigue in compromising strike mechanics is well-documented. For instance, Filimonov et al. [10] found that fatigue reduced both kicking speed and accuracy in taekwondo athletes, likely due to neuromuscular exhaustion. In boxing, fatigue has been linked to diminished limb coordination and impaired kinetic sequencing, resulting in reduced punching efficiency [11]. Similarly, balance disruption whether due to postural perturbations or external stimuli has been shown to negatively influence striking kinematics in MMA, further complicating performance under dynamic conditions [12].

The ongoing debate regarding the validity of 2D versus 3D analysis in sports biomechanics is particularly relevant in combat sports. While 3D systems remain the gold standard for capturing rotational and asymmetrical movements, previous studies have demonstrated that 2D methods can reliably assess sagittal-plane techniques such as straight punches or knee extensions [13]. In team sports like soccer and basketball, 2D tracking has been validated for jump mechanics and sprint kinematics, suggesting broader applicability [14]. However, for complex movements involving axial rotation, 3D analysis remains indispensable [15].

Recent advances in motion capture technology including artificial intelligence (AI)-assisted, markerless, and real-time tracking systems have further narrowed the gap between laboratory-grade 3D systems and practical 2D applications. These innovations offer a promising frontier in biomechanical monitoring, particularly in environments requiring both precision and portability [16].

Although previous studies have established the general validity of 2D and 3D motion capture techniques in sports biomechanics [3,5], several critical gaps remain, particularly in combat sports contexts. Most available research has focused on traditional striking disciplines such as boxing or taekwondo [7,8,10], with limited attention to MMA, where complex movement variability, dynamic postural changes, and fatigue-induced adaptations critically influence performance outcomes [17,18]. Moreover, prior comparative studies between 2D and 3D methods have primarily assessed static or controlled conditions, without evaluating biomechanical consistency under external perturbations such as balance disruption or acute fatigue [19]. Importantly, no previous research has systematically integrated variability metrics, such as the joint angle coefficient of variation (CV%), as decision criteria for the appropriate application of 2D versus 3D analysis tools in real-world athletic scenarios.

Therefore, the present study aims to fill this gap by investigating upper-limb kinematics under controlled stressors in MMA athletes and by proposing a novel variability-guided framework to optimize motion analysis strategies based on real-time task demands.

Thus, the primary objective of this study was to validate the agreement between 2D and 3D motion capture systems for measuring sagittal-plane joint kinematics during punching actions in elite MMA athletes.

The secondary objective was to examine the impact of external stressors, specifically fatigue and balance disruption, on upper-limb kinematics, joint variability, punch velocity, and impact force.

We hypothesized that (1) 2D motion analysis would show a high agreement with 3D measurements for sagittal-plane techniques, and (2) fatigue and balance disruption would impair punch mechanics by increasing joint variability, while reducing force and velocity.

## 2. Materials and Methods

### 2.1. Participants

This study included twenty-one elite male MMA fighters (mean age: 24.85 ± 7.24 years; height: 182.57 ± 6.54 cm; weight: 84.23 ± 8.87 kg), each with at least eight years of competitive experience in major organizations (UFC, Bellator, KSW). Over 80% had participated in international bouts, with most victories achieved via decision or technical knockouts (TKOs).

Eligibility criteria included consistent technical execution confirmed by licensed MMA trainers and a minimum weekly training volume of 15 h.

All participants were right-handed and performed punches using their dominant hand. Informed consent was obtained from all participants prior to this study. Only athletes without known injuries or fatigue symptoms within the preceding 48 h were included. Prior to testing, participants completed a short questionnaire confirming no engagement in strenuous activity during the preceding 24 h and confirming that they were well-rested. Eligibility criteria included consistent technical execution confirmed by licensed MMA trainers.

Additionally, all anonymized datasets, marker trajectory files, video recordings, and biomechanical metadata supporting the findings of this study have been made openly available via Zenodo (https://doi.org/10.5281/zenodo.15149188) [20]. This ensures full reproducibility and compliance with FAIR data principles.

This study adhered to the principles of the Declaration of Helsinki and was approved by the Bioethics Committee of the Academy of Physical Education in Katowice (approval number: 4/2023).

The sample size (N = 21) reflects targeted recruitment from an elite MMA population, which is inherently limited in availability.

Although modest, the sample size is consistent with previous biomechanical studies involving high-performance combat athletes.

Moreover, the high within-group homogeneity regarding training experience, handedness, and technical execution enhances internal validity and reduces variability.

Given the repeated-measures design and high measurement reliability (ICC > 0.95), this study was sufficiently powered to detect meaningful differences in joint angle parameters across varying external conditions.

While this homogeneous sampling improved internal validity, it limited the generalizability of findings to athletes of different competitive levels, genders, or lateral dominance. The inclusion of such groups in future studies could reveal distinct kinematic adaptations due to differences in neuromuscular coordination, morphological variability, or task-specific experience, potentially altering the magnitude and nature of movement variability, especially under fatigue or balance perturbation.

### 2.2. Technical Standardization and Training

Prior to testing, a standardized instruction session was conducted by an experienced MMA coach.

The session emphasized consistent punch mechanics, including stance, preparatory movement, and impact phase.

Trial sessions were performed to ensure the repeatable execution of both straight and swing punches across all experimental conditions without altering individual striking styles.

### 2.3. Motion Analysis

Reflective markers (12 mm diameter) were placed over three anatomical landmarks of the dominant upper limb: the acromion (shoulder joint center), olecranon (elbow joint center), and ulnar styloid process (wrist reference point).

All markers were positioned by the same certified biomechanist to minimize inter-operator variability. Marker positions were identical for both 2D and 3D recordings to ensure comparability.

Marker positions were identical for both 2D and 3D recordings to ensure comparability.

Calibration was performed before each testing session to confirm spatial consistency across systems.

**Figure 1:**
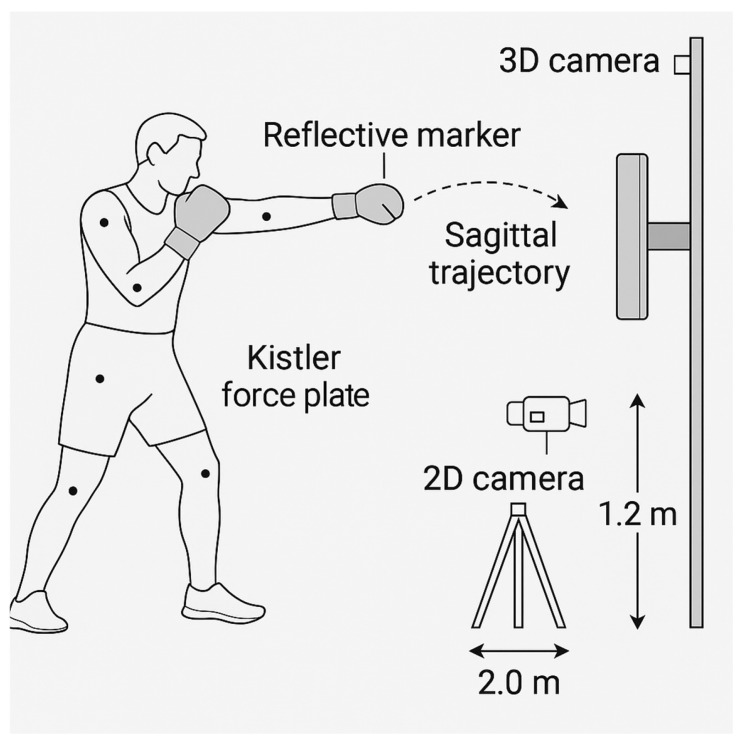
Experimental setup for 2D and 3D motion capture of sagittal-plane punches.

The schematic shows sagittal camera alignment, reflective marker positions, punch trajectory, and the vertically mounted Kistler force plate (with protective foam) (Figure 1). Shadow boxing was used in motion capture trials; impact strikes were limited to force measurement trials (not to scale).

#### 2.3.1. 2D Video Analysis

A two-dimensional analysis was performed using the my Dartfish Express app (version 7.2.0, Dartfish SA, Fribourg, Switzerland) on a Samsung Galaxy S23 Ultra (4K, 60 FPS) (Samsung Electronics Co., Ltd., Suwon, Republic of Korea), mounted on a tripod at 1.2 m height and 2.0 m distance to capture sagittal-plane motion. A detailed biomechanical description of the recorded punching sequence, including joint angles and movement phases, is provided in Supplementary Text S1, while a step-by-step outline of the experimental setup, equipment configuration, and measurement procedures is presented in Supplementary Text S2.

Marker placement details are provided in Section 2.3.

The system’s mean relative error was 3.5%, with an absolute angular error of 2.7° under optimal lighting conditions. Measurement repeatability was assessed by instructing participants to repeat each punch type three times per condition. Intra-trial reliability was evaluated using coefficient of variation (CV%) and Intraclass Correlation Coefficient (ICC) calculations. Systematic error was minimized through fixed camera positioning, standardized marker placement, and controlled lighting and background conditions.

Punch velocity was estimated frame-by-frame using high-speed video sequences (1 ms resolution) by calculating fist displacement.

A biomechanical force estimation model was employed based on hand velocity and estimated segmental mass. Segmental mass was derived using Dempster’s anthropometric tables (forearm–hand = 2.52% of body mass) [21]. The estimated kinetic energy at impact was then calculated using the classical mechanics equation, KE = ½ mv^2^, where m represents the segmental mass and v represents the velocity. Additionally, a Noraxon Ultium (Noraxon USA Inc., Scottsdale, AZ, USA) accelerometer (sampling rate: 1500 Hz), mounted on the striking glove, was used to capture acceleration data during non-contact (shadow boxing) trials. These data enabled the estimation of velocity change (Δv), which, combined with segmental mass, allowed for the calculation of impact force using the impulse–momentum relationship: F ≈ (m × Δv)/Δt. Given the short impact duration (~0.01 s), this model provides a pragmatic estimate of peak force during the terminal punch phase. While this method does not account for all degrees of freedom involved in dynamic striking, it offers a validated and practical estimation of impact force in high-speed punching movements. This dual approach, combining velocity-based and acceleration-based modeling, aligns with previous studies in combat sports biomechanics [17,18], and enabled the indirect estimation of impact kinetics for comparison with direct force measurements obtained via the Kistler force plate in selected trials.

It offers a practical surrogate for direct force measurements when working with 2D systems lacking ground reaction data and has been previously shown to yield valid estimates of striking force based on end-effector velocity and known segmental mass.

#### 2.3.2. 3D Motion Analysis

Three-dimensional analysis was conducted using the MaxPRO motion capture system (Innovision Systems Inc., Columbiaville, MI, USA) infrared camera system (4 cameras, 240 Hz, mounted at 2.5 m), offering a spatial accuracy of 0.5 mm and angular error of less than 2%.

The system was calibrated before each session using both static and dynamic protocols recommended by the manufacturer.

Marker placement mirrored that described in Section 2.3.

Trajectories were post-processed using low-pass Butterworth filtering (10 Hz), and time-series alignment ensured temporal consistency.

In selected trials focused on impact measurement, participants delivered punches into a vertically mounted Kistler 9287CA force plate padded with 5 mm of high-density foam. These contact-based trials were conducted independently of the motion capture sessions and were used to validate force models derived from non-contact recordings.

To assess repeatability, ICC values were computed across repeated trials for each joint and punch type.

Punch velocity and impact force were recorded in separate contact-based trials using a vertically mounted Kistler 9287CA (Kistler Instrumente AG, Winterthur, Switzerland), force plate (1000 Hz) and a Noraxon Ultium (Noraxon USA Inc., Scottsdale, AZ, USA) accelerometer (1500 Hz) attached to the glove.

Impact force was calculated as the resultant vector magnitude derived from three orthogonal components (Fx, Fy, Fz) using the formula √(Fx^2^ + Fy^2^ + Fz^2^).

### 2.4. Experimental Procedures

Participants performed three straight and three swing punches under three experimental conditions:

- Normal—standard stance without perturbation.
- Balance Disruption—induced by 30 s of rapid spinning to induce transient vestibular disorientation, simulating acute postural instability encountered after grappling exchanges, takedowns, or unanticipated body rotation during real combat situations.
- Fatigue—induced by 10 high-intensity bodyweight drills.

Fatigue was induced using a standardized high-intensity protocol consisting of 10 bodyweight drills (burpees, jump squats, mountain climbers) performed in rapid succession with minimal rest, lasting approximately 90–120 s. This protocol was selected based on previous studies validating its efficacy in inducing acute neuromuscular fatigue in combat athletes. Completion was supervised by a certified trainer to ensure full compliance. Fatigue was verified subjectively using a verbal confirmation of perceived exertion (RPE ≥ 8 on the Borg CR10 scale) immediately after the drill set. Objectively, a visible decline in movement speed and technique sharpness during the final repetitions was used as a secondary indicator by the supervising coach. This dual-method approach ensured consistent fatigue levels across participants.

A standardized two-minute rest period was applied between conditions.

Straight punches (SRPs) were executed strictly along the sagittal plane with minimal trunk rotation.

Swing punches (SPs) followed a diagonal trajectory, combining sagittal and transverse plane components to simulate a hook motion.

All punches were performed at maximal voluntary intensity following a verbal command.

No physical targets were struck during motion capture trials (i.e., shadow boxing), in order to avoid marker displacement and maintain tracking consistency. However, in a subset of force measurement trials, athletes delivered punches into a vertically mounted Kistler force plate covered with high-density foam (5 mm).

Participants were instructed to prioritize speed, technical precision, and biomechanical alignment.

### 2.5. Data Collection Environment

Testing was conducted in a controlled biomechanics laboratory where lighting, temperature, and humidity were standardized to ensure measurement reliability, especially for 2D analysis, where visual clarity is critical.

Camera positions were stabilized to eliminate parallax and vibration. Pre-trial readiness was confirmed through a brief dynamic warm-up and verbal confirmation of no acute fatigue. If signs of abnormal fatigue or discomfort were present, the session was rescheduled.

During kinematic analyses, participants performed punches in air (shadow boxing) to avoid marker displacement. However, for select trials involving force measurements, athletes delivered punches into a vertically mounted Kistler force plate padded with high-density foam (5 mm). This setup allowed impact force to be recorded without compromising marker visibility or safety.

Punches were directed toward the mounted plate while wearing standardized 10 oz MMA training gloves.

All raw kinematic and force data are publicly available via Zenodo (https://zenodo.org/, accessed on 15 May 2025) [20].

### 2.6. Data Reduction and Processing

Joint angles were calculated in the sagittal plane relative to a static anatomical calibration posture. The anatomical position, defined as the arm fully extended alongside the torso (0°), served as the reference frame. Elbow flexion angles increased with joint bending, where full extension corresponded to 0° and maximal flexion during punches ranged between 45° and 90°. Shoulder abduction was also defined as a positive angular change from the neutral axis, with values typically ranging from 10° to 30° at peak punch extension. These conventions were applied consistently across both 2D and 3D systems to ensure comparability of angular measurements.

For consistency with 2D analysis, 3D joint angles were extracted from the sagittal plane of the global reference frame, based on laboratory coordinate axes. No segment-based local reference frames were used in angular calculations.

For the 3D system, marker trajectories were low-pass filtered (Butterworth, second-order, 6 Hz cutoff), and joint angles were calculated based on standardized segment definitions (acromion, olecranon, ASIS).

Punch velocity was calculated from high-speed video data by measuring fist displacement over the final 10 ms preceding impact. Peak impact force was extracted from force plate recordings by identifying the maximum force value within the striking window.

Artifacts such as marker occlusion or motion blur were screened, and affected trials were repeated. For each punch type and condition, the mean of three valid repetitions was used for analysis. Angular data were normalized relative to the initial anatomical calibration posture (arm fully extended alongside the torso), to ensure valid comparison across measurement systems.

### 2.7. Statistical Methods

The dependent variables analyzed in this study are as follows:

- Elbow joint angle (°)—determined from sagittal-plane markers (olecranon, ulnar styloid) captured via 2D and 3D motion capture.
- Shoulder joint angle (°)—determined from sagittal-plane markers (acromion, olecranon) captured via 2D and 3D motion capture.
- Punch velocity (m/s)—estimated frame-by-frame from fist displacement over time using high-speed video (1 ms resolution) and accelerometry data.
- Impact force (N)—recorded directly via Kistler 9287CA force plate (floor-mounted, 1000 Hz sampling rate).
- Coefficient of variation (CV%) for joint angles and impact force—calculated as (standard deviation/mean) × 100 to assess intra-individual variability across trials.

Each dependent variable was measured across three experimental conditions (normal, balance disruption, fatigue) and for two punch types (straight punch, swing punch).

Data integrity was assessed for outliers using boxplot inspection and the 1.5 IQR rule.

Normality was verified with the Shapiro–Wilk test (*p* > 0.05 for all conditions), and homogeneity of variances was tested using Levene’s test. The results indicated non-significant variance differences across all comparisons (*p* > 0.05), confirming the suitability of one-way ANOVA assumptions. This ensured that variance homogeneity was not violated in the analysis of joint angle data under different external conditions.

Descriptive statistics (mean, SD, median, IQR) were computed for each condition.

Pearson’s correlation coefficient was used to assess agreement between 2D and 3D measures.

Group comparisons were performed using one-way ANOVA, followed by Tukey’s HSD post hoc test for pairwise differences. The independent variables in the ANOVA models were condition (normal, vestibular disruption, fatigue) and punch type (straight vs. swing). Dependent variables included joint angles, punch velocity, impact force, and CV%.

Intraclass Correlation Coefficient (ICC) quantified the intra-individual reliability of angular data.

To model individual variability in punch mechanics across conditions, a linear mixed model (LMM) was employed, with fatigue, balance, and punch type as fixed effects, and subjects as random effects.

The coefficient of variation (CV%) was calculated for joint angle consistency and impact force, as recommended in previous biomechanical variability assessments [20].

To ensure repeatability, each punch condition was repeated three times and analyzed independently. Intraclass Correlation Coefficients (ICC 2,k) were calculated to assess the consistency of angular measurements across trials. For 2D analysis, systematic errors were controlled by enforcing fixed camera position, controlled lighting, and precise marker placement by the same operator. In 3D analysis, system calibration was performed before each session, and trajectories were filtered and aligned to prevent temporal drift. No significant systematic drift was detected between trials (*p* > 0.05, repeated-measures ANOVA). The overall intra-session repeatability exceeded ICC = 0.90 across both methods.

To evaluate practical equivalence between the 2D and 3D methods for sagittal-plane joint angle measurement, we established an a priori equivalence margin of ±1.5°, based on previous validation studies of angular accuracy in sport motion capture systems [21,22]. Any mean absolute difference below this threshold was interpreted as an acceptable level of measurement agreement for training purposes.

All analyses were conducted using STATISTICA 13.1 (StatSoft Inc., Tulsa, OK, USA), and post hoc power analysis was validated using GPower 3.1 software (Heinrich-Heine-University Düsseldorf, Germany).

Post hoc statistical power analyses confirmed sufficient sensitivity to detect large and moderate effects (f ≥ 0.25) in the one-way ANOVA and linear mixed models. Observed power values (1 − β) for the main comparisons exceeded 0.84, indicating acceptable statistical power for hypothesis testing given the sample size and within-subjects design.

## 3. Results

Levene’s test confirmed equal variances across conditions (*p* > 0.05), supporting the use of parametric tests.

### 3.1. Validation of 2D and 3D Motion Capture Systems

Table 1 presents the correlation analysis between elbow joint (EJ) and shoulder joint (SJ) angles recorded by 2D and 3D systems during straight right punches (SRPs) and swing punches (SPs). For each punch type and condition, joint angles were calculated at the moment of peak arm extension (i.e., maximal reach prior to follow-through). Each participant completed three repetitions per punch type and condition; angular data were averaged across the three trials before statistical analysis. Correlations between 2D and 3D measurements were based on sagittal-plane kinematics extracted from the 3D system to match the 2D projection plane.

The correlations between 2D and 3D measurements were consistently high, ranging from r = 0.964 to 0.999. The highest correlation was observed for the shoulder joint during swing punches (SP-SJ: r = 0.999), while the lowest was noted for the elbow joint during swing punches (SP-EJ: r = 0.964). Strong agreement was also found for the elbow joint during straight punches (SRP-EJ: r = 0.992).

Joint angles were consistently measured in the sagittal plane for both 2D and the sagittal projection of 3D data to ensure methodological consistency. For each punch, the maximum joint extension angle (i.e., maximal forward reach) was extracted. The angles reported in Table 2, Table 3 and Table 4 represent mean values averaged across three valid repetitions for each participant under each condition.

Table 2, Table 3 and Table 4 summarize the joint angle comparisons and corresponding Intraclass Correlation Coefficient (ICC) values across three experimental conditions: normal, vestibular disruption, and fatigue.

Under vestibular disruption (Table 3), ICC values ranged from 0.897 to 0.944, indicating high consistency between the two measurement methods despite induced balance perturbations. The lowest ICC was recorded for the shoulder joint during SRPs (0.897), while the highest was observed for the shoulder joint during SPs (0.944).

Under fatigue conditions (Table 4), ICC values remained robust (0.925–0.974), demonstrating that physical exertion did not significantly compromise measurement agreement. The highest reliability was again found for the shoulder joint during swing punches (ICC = 0.974).

**Figure 2:**
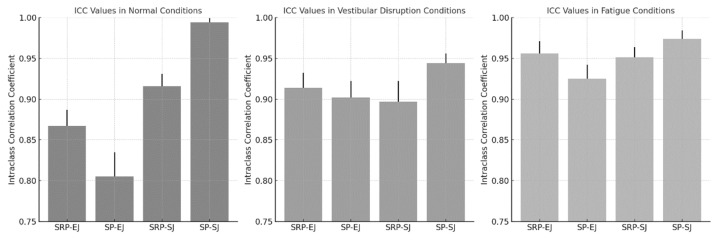
Intraclass Correlation Coefficient (ICC) values for elbow and shoulder joint angles during straight right punches (SRPs) and swing punches (SPs) across three experimental conditions: normal, vestibular disruption, and fatigue. Error bars represent standard deviation.

Figure 2 visualizes ICC values across punch types and experimental conditions. As shown, ICC values remained consistently high under all conditions, ranging from 0.805 to 0.994 in the normal condition, 0.897 to 0.944 during vestibular disruption, and 0.925 to 0.974 under fatigue. Shoulder joint measurements consistently demonstrated higher reliability compared to the elbow joint.

This disparity may be attributed to the biomechanical characteristics of each joint. The shoulder joint typically exhibits more stable and constrained movement patterns during sagittal-plane punching, especially in well-trained athletes. In contrast, the elbow joint is more susceptible to minor deviations due to its role in dynamic force transfer and its higher degrees of freedom in flexion–extension. These factors may introduce slightly greater intra-individual variability in elbow joint kinematics, thereby reducing ICC values despite overall high reliability.

**Figure 3:**
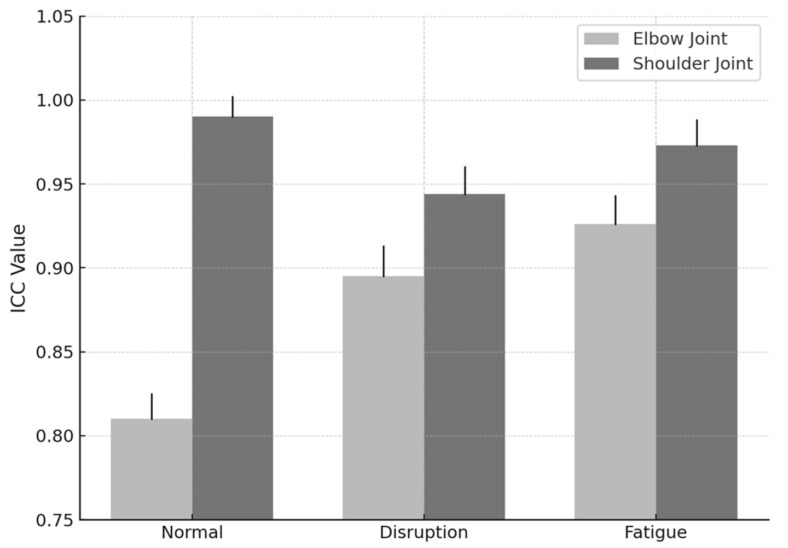
Mean Intraclass Correlation Coefficient (ICC) values for elbow and shoulder joints across three experimental conditions (normal, balance disruption, and fatigue). Data represent average ICC scores across both punch types (straight right punch and swing punch). Error bars indicate standard deviation. Shoulder joints demonstrated consistently higher reliability across all conditions.

To further illustrate joint stability, Figure 3 presents mean ICC values aggregated across all joints and conditions.

**Figure 4:**
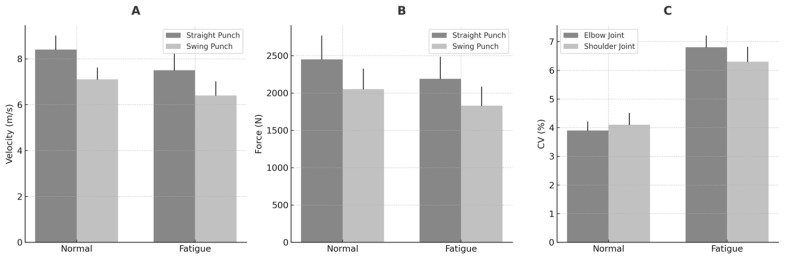
(**A**–**C**): Biomechanical performance metrics under normal and fatigue conditions. (**A**) Mean punch velocity (m/s); (**B**) peak impact force (N); (**C**) coefficient of variation (CV%) for elbow and shoulder joint angles. Fatigue was associated with significant declines in velocity and force and increased kinematic variability. Data represent mean values across three trials per condition; error bars indicate standard deviation.

To visualize the effects of fatigue on key performance metrics, Figure 4 presents a panel-based summary of changes in punch velocity (A), impact force (B), and joint angle variability (CV%) across the elbow and shoulder joints (C).

### 3.2. Biomechanical Effects of Fatigue and Balance Disruption

Punch velocity and impact force were also significantly affected by fatigue. Under normal conditions, mean punch velocities were 8.4 ± 0.6 m/s for straight punches and 7.1 ± 0.5 m/s for swing punches. Under fatigue, velocities declined significantly to 7.5 ± 0.7 m/s and 6.4 ± 0.6 m/s, respectively (*p* < 0.01).

Similarly, the peak impact force decreased from 2450 ± 315 N to 2190 ± 290 N for straight punches, and from 2050 ± 270 N to 1830 ± 250 N for swing punches.

Kinematic variability also increased under fatigue, as reflected by higher coefficients of variation (CV%). Shoulder joint CV increased from 4.1% (normal) to 6.3% (fatigue; *p* = 0.003), while elbow joint CV rose from 3.9% to 6.8% (*p* = 0.002). These results suggest that neuromuscular fatigue impairs motor control and reduces joint stability.

Linear mixed model (LMM) analysis confirmed significant effects of fatigue on punch velocity (F(1,20) = 12.45, *p* = 0.002) and impact force (F(1,20) = 10.82, *p* = 0.004). Inter-individual variability accounted for approximately 21% of the total variance, emphasizing the importance of personalized training and monitoring.

While this analysis did not explicitly model individual-level covariates, the observed variance may be partially attributed to differences in punching technique, neuromuscular efficiency, or biomechanical strategies developed through varied training histories. Elite athletes often exhibit idiosyncratic adaptations in joint coordination and movement tempo, even under standardized task constraints. Future studies incorporating electromyography (EMG) and motion complexity metrics may help disentangle how specific athlete characteristics contribute to inter-individual variability.

Across all conditions, the mean angular difference between 2D and 3D measurements remained consistently below 1° (absolute mean difference: 0.68° ± 0.14°). Neither fatigue (*p* = 0.742) nor balance disruption (*p* = 0.689) significantly affected measurement agreement.

These differences fell well within the predefined equivalence margin of ±1.5°, indicating that from a practical standpoint, both systems provided sufficiently interchangeable measurements for assessing joint angles in sagittal-plane techniques.

## 4. Discussion

This study primarily aimed to validate the agreement between 2D and 3D motion capture systems in assessing sagittal-plane punching kinematics. Our results demonstrated strong correlations (r = 0.964–0.999) and high reliability (ICC = 0.81–0.99) across both systems, confirming that 2D video analysis provides sufficient precision for evaluating fundamental punching techniques in MMA athletes under varying external conditions.

In addition to this validation objective, the study investigated the effects of fatigue and balance disruption on upper-limb biomechanics. As expected, external perturbations impaired punch mechanics by reducing velocity, impact force, and joint stability, consistent with theoretical models of neuromuscular fatigue and adaptive motor control [23,24].

Video-based analysis has become an essential tool in sports biomechanics, enabling the precise evaluation of movement patterns and facilitating performance optimization. Both 2D and 3D motion capture systems have been extensively utilized to assess athletic techniques. While 3D analysis offers superior accuracy in tracking multi-planar motion and joint rotations, 2D analysis remains a widely accessible and cost-effective alternative [18]. However, the primary limitation of 2D methods lies in their inability to capture depth and subtle biomechanical variations, which are crucial for evaluating complex techniques such as rotational kicks and throws [3,25].

The findings of this study can also be interpreted within the broader context of motor control theories emphasizing the role of structured variability [15,16]. The minor increases in joint angle variability (CV%) observed under fatigue conditions suggest an adaptive, rather than purely detrimental, response. According to variability performance relationship models [26], optimal variability allows for the preservation of critical task-relevant variables (e.g., fist trajectory) while accommodating physiological perturbations [15]. In this view, the increased elbow joint variability observed in fatigued conditions may reflect flexible motor strategies aimed at maintaining punch effectiveness rather than an outright degradation of technique. This interpretation supports the notion that elite MMA athletes possess refined compensatory mechanisms enabling stable performance under adverse conditions [2,12,27].

This study further reinforces the existing literature by confirming that 2D analysis is highly reliable for assessing fundamental striking techniques, even under challenging conditions such as fatigue and balance disruption. The correlation values observed (r > 0.96) indicate that 2D motion capture yields nearly identical measurements to those of 3D systems for straight and swing punches. These findings align with prior studies demonstrating the sufficiency of 2D tracking for sagittal-plane kinematics evaluation in combat sports [3] and extend to comparable results observed in modalities such as soccer passing mechanics, gymnastic landings, treadmill running, and repeated sprinting tasks, where movement occurs primarily in constrained sagittal planes and is characterized by reproducible biomechanical patterns, even under fatigue, supporting high inter-method agreement [28,29,30,31].

Importantly, we did not rely solely on correlation coefficients to establish methodological agreement. A predefined equivalence threshold of ±1.5° was applied, and the observed mean absolute differences between 2D and 3D angles (0.68° ± 0.14°) consistently remained below this margin, supporting the practical interchangeability of the two methods for routine assessments.

While the use of correlation coefficients and ICC provided strong evidence of consistency, we recognize that these metrics alone do not confirm methodological equivalence. Although we applied an a priori equivalence threshold (±1.5°), future studies should consider formal statistical equivalence testing, such as the two one-sided tests (TOST) procedure or Bland–Altman analysis, to provide more robust confirmation. The present study’s small mean angular deviation and high ICCs (>0.90) supported our conclusions, but we acknowledge the added value of equivalence testing in future validation efforts.

This study also revealed higher agreement for shoulder joint (SJ) angles (ICC = 0.99) compared to elbow joint (EJ) angles (ICC = 0.81–0.96), likely reflecting the more stable movement patterns of the shoulder. In contrast, elbow joints exhibited slightly lower agreement, potentially due to greater variability in flexion–extension dynamics [5]. Interestingly, fatigue conditions yielded higher ICC values (0.95–0.99) than either normal (0.81–0.99) or vestibular-disruption conditions (0.84–0.98). This suggests that athletes, under fatigue, may adopt more controlled and deliberate punching techniques to maintain efficiency, thereby reducing execution variability a finding consistent with previous investigations into fatigue effects on motor control [2].

The choice of rapid spinning as a method to induce balance disruption was based on its validated ability to create transient vestibular disorientation under controlled conditions. While simplified, this approach simulates postural instability frequently experienced in real-world MMA bouts such as during clinch disengagements, rotational throws, or unexpected body torques. It provides a reproducible, safe, and ethically viable way to induce balance perturbations without compromising athlete safety. We acknowledge that this method does not replicate the full spectrum of multi-planar disturbances present during live combat. However, it remains an experimentally justified proxy for evaluating upper-limb motor control under vestibular challenge.

Although the increased ICC under fatigue suggests improved internal consistency, it may also reflect a dual mechanism: (1) individual compensatory strategies adopted to preserve performance, and (2) a potential simplification or stereotyping of movement patterns under neuromuscular constraint. Elite fighters often adopt stable, repetitive motor solutions to maintain execution quality, which could reduce variability but limit adaptive flexibility. Previous research has demonstrated such kinematic adaptations during fatigue, including altered joint stiffness, trunk inclination, and modified punch sequencing [2,8,16]. While the present study did not explicitly quantify these compensatory strategies, the linear mixed model (LMM) analysis revealed that individual differences accounted for 21% of the total variance in punch execution. This variance likely reflects athlete-specific technical styles, neuromuscular coordination strategies, or habitual motor patterns despite uniform elite status and extensive training experience. This finding underscores the need for future research exploring individual adaptation profiles, potentially employing machine learning approaches to classify and predict compensation mechanisms.

Building upon these findings, we propose a hybrid analytical framework wherein the choice between 2D and 3D motion analysis is dynamically guided by observed variability in joint kinematics. Specifically, when sagittal-plane joint angle variability remains below a 5% threshold (CV%), 2D analysis may be considered sufficient for routine monitoring. Conversely, when variability exceeds this threshold particularly under fatigue or perturbation conditions, 3D analysis becomes necessary to capture complex multi-planar adaptations. This adaptive strategy, which we term the Variability-Guided Motion Capture Decision Framework, promotes an efficient allocation of biomechanical resources and tailors analytical depth to task-specific demands.

Furthermore, the observed increases in joint angle variability (CV%) and reductions in impact force under fatigue highlight important biomechanical mechanisms. Elevated CV% values in the elbow and shoulder joints likely reflect compromised intersegmental coordination, disrupting the efficient transfer of kinetic energy along the kinetic chain [28]. Consequently, reduced punch velocity and impact force can be interpreted as functional indicators of neuromuscular fatigue impairing proximal-to-distal segmental sequencing, a critical factor in optimal striking performance [29]. These results suggest that the biomechanical monitoring of CV% and force metrics could serve not only as performance diagnostics but also as early markers of motor control deterioration during high-intensity combat sports activities [30].

Although the angular differences between 2D and 3D methods were numerically small (<1°), their biomechanical relevance should not be underestimated. Previous studies have demonstrated that even minor deviations in joint angles, particularly at the elbow and shoulder, can influence punch effectiveness. For example, Cheraghi et al. [11] reported that elbow angle variations of 2–3° significantly affect impact force and punch velocity in professional boxers. Similarly, Pozo et al. [12] highlighted the critical role of shoulder positioning in maximizing kinetic chain efficiency. Comparable compensatory patterns and high ICC values under fatigue have also been observed in lower-limb movements such as vertical jumping and treadmill running, supporting the generalizability of neuromuscular adaptation strategies across sports contexts [32,33]. Therefore, the high ICC and correlation values observed in our study confirm not only statistical agreement but also the practical equivalence of 2D and 3D methods in detecting performance-relevant biomechanical changes.

These observations directly support the study’s primary aim: to evaluate how external factors specifically fatigue and balance disruption influence upper limb kinematics during punching techniques. The greater inter-joint variability observed at the elbow compared to the shoulder highlights the elbow’s sensitivity to external perturbations and neuromuscular fatigue. This suggests that distal segments of the upper limb may require targeted control strategies to preserve technical integrity under stressful conditions. Understanding such joint-specific adaptations provides valuable insights for combat sport practitioners aiming to optimize striking mechanics in unstable or fatigue-prone contexts.

From a practical standpoint, the present findings suggest that 2D video analysis can be effectively employed by coaches and therapists in field-based settings to monitor joint kinematics during striking drills, provided that sagittal-plane techniques are used and standardized procedures are followed. Using widely available tools such as smartphone cameras and motion analysis apps, practitioners can track joint angles, assess consistency, and detect deviations from optimal technique over time. This can facilitate early identification of fatigue-induced compensations, technical errors, or asymmetries, thereby supporting targeted intervention without requiring high-cost 3D infrastructure. The use of a CV% threshold (5%) may also serve as a practical benchmark for deciding when deeper biomechanical assessment is warranted.

The integration of both 2D and 3D motion analysis methods offers complementary advantages for athlete monitoring in combat sports. We envision a tiered protocol, in which 2D video analysis serves as a first-line tool for routine technique monitoring, especially in field settings, thanks to its accessibility and portability. In contrast, 3D systems may be selectively deployed for in-depth assessments, such as during performance diagnostics, injury risk evaluations, or when multi-planar movements are of interest. This layered approach could be embedded within long-term athlete development frameworks, enabling continuous feedback cycles and the individualized adjustment of training strategies [18]. This complementary approach enables more individualized training strategies, ensuring maximal biomechanical efficiency across varied physiological and perceptual conditions. To operationalize this integration in practice, we propose a tiered implementation model. In this framework, 2D video analysis leveraging smartphone-based or tablet-based tools would serve as a frontline assessment tool during regular training sessions, allowing coaches to quickly identify deviations from baseline technique or increased movement variability. When these deviations exceed predefined thresholds (CV% > 5%), athletes could be referred for 3D motion capture evaluations to explore underlying multi-planar compensations in greater depth. This cascade approach enables scalable monitoring, reserving 3D analysis for cases that require high-resolution kinematic insight, such as performance diagnostics, rehabilitation, or return-to-play decisions. Importantly, digital documentation from 2D systems can inform the selection and timing of 3D assessments, creating a closed-loop feedback cycle that enhances the precision and efficiency of individualized technical training. Moreover, given the growing availability of portable sensor technologies such as high-speed smartphone cameras, wearable inertial measurement units (IMUs), wireless EMG systems, and computer vision-based markerless tracking tools, future applications may increasingly incorporate multi-sensor platforms for real-time biomechanical analysis. Such technologies offer significant potential for capturing motion data outside laboratory settings, facilitating in-field performance diagnostics and feedback systems in combat sports.

Several external factors may influence the accuracy of motion analysis measurements. Laboratory lighting conditions, for instance, can affect marker detection precision in 2D analysis, particularly during manual angle determinations [34]. Furthermore, even with careful calibration, minor discrepancies in 2D and 3D camera positioning may introduce measurement errors such as blind spots or marker occlusions. These technical limitations highlight the need for robust sensor fusion algorithms and standardized calibration protocols. Future research should address these challenges by validating measurement frameworks across diverse sport-specific environments and leveraging machine learning techniques to enhance accuracy and automate data processing.

As motion analysis technology continues to evolve, advancements such as the miniaturization of motion-capture devices, integration of deep learning-based pose estimation (Open Pose, Media Pipe), and wearable sensor networks are expected to enhance both the precision and scalability of biomechanical monitoring. The current findings contribute to this trajectory by demonstrating that 2D video analysis remains a valid and reliable tool for upper limb movement assessment, even under challenging external conditions such as fatigue and vestibular disruption. Future studies should explore the feasibility of AI-driven, markerless tracking systems for real-time punch analysis during sparring or competition, which could significantly support performance diagnostics, motor learning, and injury prevention in combat sports.

It should be noted that 3D joint angles were extracted relative to the global reference frame, matching the sagittal projection used in 2D analysis. While this approach ensures direct comparability between systems, it does not exploit the full potential of 3D kinematic analysis, such as local segment-based joint angle computation or multiplanar motion tracking. Consequently, the present results demonstrate that sagittal-plane global angles are comparable between 2D and 3D systems under controlled conditions, but this does not imply that 2D analysis is suitable for complex multi-planar techniques where out-of-plane rotations and segment-relative measurements are critical.

Furthermore, integration with mobile platforms such as smartphones and tablets equipped with embedded cameras and IMUs could enable on-site video analysis and feedback for athletes and coaches. Artificial intelligence algorithms, including convolutional neural networks (CNNs) and recurrent neural networks (RNNs), could be employed to automatically detect suboptimal joint positions, classify punch types, or predict injury risk based on deviations in kinematic patterns. Such innovations could play a transformative role in personalized performance monitoring, rehabilitation, and adaptive coaching systems.

## 5. Conclusions

This study demonstrates that 2D motion analysis is a reliable, valid, and accessible method for evaluating fundamental striking mechanics in elite MMA athletes. Our findings revealed strong agreement between 2D and 3D systems (ICC = 0.81–0.99; r = 0.964–0.999), confirming that 2D analysis provides sufficient precision for assessing sagittal-plane punch kinematics across various external conditions. Given its high concordance with 3D motion capture and its ease of use, 2D analysis appears particularly well-suited for routine biomechanical monitoring, provided that standardized protocols are followed.

While 3D systems remain the gold standard for capturing multi-planar motion and subtle joint asymmetries, 2D analysis offers substantial value in practical training contexts. The complementary use of both 2D and 3D systems is supported by the observed inter-individual variability (accounting for 21% of total variance), suggesting the utility of combining these methods for individualized technique monitoring, particularly under conditions of fatigue-induced kinematic instability (CV% increase: *p* < 0.01).

These conclusions are directly supported by the statistical equivalence of joint angle measurements across all tested conditions and the minimal absolute difference between 2D and 3D methods (mean error < 1°), further highlighting the practical viability of 2D systems in real-world combat sports settings. Importantly, the validated reliability of 2D analysis under perturbed conditions suggests its potential integration into real-time feedback systems during training sessions. When paired with mobile technologies or markerless tracking algorithms, 2D video analysis could facilitate on-site performance diagnostics, enabling coaches to provide immediate technical corrections based on sagittal-plane kinematic data. Such applications may enhance motor learning and injury prevention without the need for complex laboratory infrastructure. However, as all measurements were performed in a laboratory setting, the generalizability of our findings to field conditions—such as live sparring or competition—remains to be tested. Future studies should validate the robustness of 2D motion capture under dynamic, real-world environments.

Future studies are encouraged to validate these findings across more complex punch trajectories and diverse athlete populations.

## 6. Limitations

Despite its contributions, this study has several methodological limitations that should be acknowledged.

First, the exclusive focus on straight and swing punches, although methodologically robust, limits the extrapolation of findings to more complex, multi-planar techniques such as rotational kicks or grappling transitions. Consequently, the observed reliability of 2D analysis may not extend to movements involving higher degrees of axial rotation.

Second, this study was conducted in a controlled laboratory environment. While this setting ensured standardization, it may not fully replicate the real-world variability associated with visual feedback, opponent interactions, and spontaneous execution, all of which could influence arm positioning and joint dynamics during live competition.

Third, the sample was composed exclusively of elite, right-handed male fighters, resulting in reduced inter-individual variability. Although this homogeneity enhanced internal validity, it potentially inflates reliability estimates. In more diverse populations, similar levels of 2D–3D agreement may not be achievable.

Fourth, small errors in marker placement and camera calibration particularly in 2D video analysis may have introduced systematic measurement bias, especially regarding elbow joint angles where slightly lower ICC values were observed. These technical factors could partially explain the variability in agreement between joints and across experimental conditions.

Finally, although the sample size aligns with previous biomechanical studies in combat sports, it may limit the statistical power for detecting subtle condition-specific effects. Future research should employ larger and more heterogeneous samples and incorporate calibration error quantification to better isolate systematic sources of variability.

An additional limitation is the lack of direct assessment regarding participants’ prior familiarity with video-based motion analysis tools. Although all athletes were experienced professionals likely accustomed to performance feedback through video analysis, future studies should formally control for this factor.

Further investigations may also consider applying formal equivalence testing techniques, such as Bland–Altman plots or two one-sided tests (TOSTs), to more rigorously evaluate agreement between 2D and 3D methods.

Although post hoc power analyses indicated sufficient sensitivity to detect the primary effects, future research should aim for larger and more diverse cohorts to ensure adequate power for identifying smaller, interaction-level effects.

## 7. Future Directions in Motion Analysis for Combat Sports

Future research should prioritize the implementation of real-time biomechanical feedback systems during sparring and live competition settings. Technologies such as wearable inertial measurement units (IMUs), AI-based pose estimation systems, and markerless tracking platforms offer promising, unobtrusive, and scalable solutions for real-world biomechanical analysis [15,17].

Another important research avenue involves formalizing the Variability-Guided Motion Capture Decision Framework into a fully algorithmic decision-making model. Leveraging real-time variability metrics collected via wearable or markerless systems, such models could dynamically switch between 2D and 3D analysis modes based on the athlete’s neuromuscular state and the complexity of the task, thereby optimizing performance diagnostics in the field.

Analyzing inter-individual variability across different experience levels and fighting styles constitutes another key direction. For example, comparing striking mechanics between novice, intermediate, and elite athletes may uncover critical motor control adaptations, as proposed by Davis et al. [14] and Sheikhhoseini et al. [8].

From a technological standpoint, future efforts should focus on developing automated software capable of extracting reliable biomechanical data from standard video footage. Machine learning-based systems could identify movement inefficiencies in real time, facilitating immediate technique refinement and contributing to injury prevention strategies [34].

By integrating these technological innovations, future research can help bridge the existing gap between laboratory-based motion analysis and real-world athletic training, advancing evidence-based practice in MMA and other high-intensity sports contexts.

## Figures and Tables

**Table 1:** Correlation coefficients between 2D and 3D joint angle measurements during SRP and SP.

Variables	SRP-EJ (3D)	SP-EJ (3D)	SRP-SJ (3D)	SP-SJ (3D)
SRP-EJ (2D)	0.992			
SP-EJ (2D)		0.964		
SRP-SJ (2D)			0.998	
SP-SJ (2D)				0.999

Note: EJ—elbow joint; SJ—shoulder joint.

**Table 2:** Comparison of joint angles and ICC values under normal conditions.

Punch Type and Joint	Angle (°)—2D	Angle (°)—3D	ICC
Straight Right Punch–Elbow Joint	51.86	51.41	0.867
Swing Punch–Elbow Joint	48.77	49.56	0.805
Straight Right Punch–Shoulder Joint	24.30	23.82	0.916
Swing Punch–Shoulder Joint	12.77	12.81	0.994

**Table 3:** Joint angle comparison and ICC values during vestibular disruption.

Punch Type and Joint	Angle (°)—2D	Angle (°)—3D	ICC
Straight Right Punch–Elbow Joint	51.43	51.14	0.914
Swing Punch–Elbow Joint	53.34	53.03	0.902
Straight Right Punch–Shoulder Joint	18.29	17.63	0.897
Swing Punch–Shoulder Joint	11.78	11.39	0.944

**Table 4:** Comparison of joint angle measurements in fatigue conditions.

Punch Type and Joint	Angle (°)—2D	Angle (°)—3D	ICC
Straight Right Punch–Elbow Joint	55.89	56.06	0.956
Swing Punch–Elbow Joint	49.72	49.42	0.925
Straight Right Punch–Shoulder Joint	25.84	25.53	0.951
Swing Punch–Shoulder Joint	13.20	13.02	0.974

## Data Availability

The dataset supporting this study is publicly available at Zenodo: https://doi.org/10.5281/zenodo.15149188 [20].

## Supplementary Materials

The following supporting information can be downloaded at: https://www.mdpi.com/article/10.3390/s25113270/s1, Supplementary Text S1; Supplementary Text S2.

## Abbreviations

The following abbreviations are used in this manuscript:

MMA	Mixed Martial Arts
2D	Two-Dimensional
3D	Three-Dimensional
ICC	Intraclass Correlation Coefficient
CV%	Coefficient of Variation (Percentage)
SRP	Straight Right Punch
SP	Swing Punch
EJ	Elbow Joint
SJ	Shoulder Joint
LMM	Linear Mixed Model
IMU	Inertial Measurement Unit
EMG	Electromyography
AI	Artificial Intelligence
CNN	Convolutional Neural Network
RNN	Recurrent Neural Network
TKO	Technical Knockout
FPS	Frames Per Second
SD	Standard Deviation
ANOVA	Analysis of Variance
HSD	Honestly Significant Difference
Q–Q	Quantile–Quantile Plot
APA	American Psychological Association
