# Supplementary material for: Evaluation of the Impact of External Conditions on Arm Positioning During Punches in MMA Fighters: A Comparative Analysis of 2D and 3D Methods
Source: Sensors (Basel). 2025 May 22;25(11):3270. doi: 10.3390/s25113270 (PMC12157019; doi:10.3390/s25113270)
Supplement: Supplementary file 1 [file sensors-25-03270-s001.zip › sensors-3600233-supplementary.pdf]

Supplementary Text S1.

Biomechanical Description of Punch Execution Captured via 2D Motion Analysis

The analyzed striking sequence consisted of three distinct kinematic phases: preparation, impact, and follow-through, recorded using a standardized two-dimensional video setup.

During the preparation phase, the participant adopted a stance characterized by approximately 75° to 80° of shoulder abduction relative to the torso and about 90° of elbow flexion. The wrist was maintained in a neutral alignment (0° flexion/extension and 0° pronation/supination). The center of mass was positioned slightly posterior to the midline of support to preload the kinetic chain and optimize force transmission. Reflective markers were securely positioned on the acromion and olecranon landmarks to ensure precise sagittal-plane tracking of upper-limb segments. The preparatory motion involved a slight posterior retraction of the shoulder girdle combined with elbow flexion, priming the musculature for subsequent explosive extension.

In the impact phase, maximal forward extension of the upper limb was achieved. Shoulder abduction increased to approximately 90°, with elbow extension reaching nearly full range (170°–175°), facilitating efficient kinetic energy transfer through the arm to the target. The wrist orientation was maintained in slight pronation (~10°–15°) to align the forearm and fist along the sagittal axis and maximize striking surface stability. The reflective markers exhibited near-linear trajectories, indicating minimal angular deviation within the sagittal plane. Frame-by-frame analysis demonstrated peak fist velocity ranging from 7.5 to 8.4 meters per second under normal, non-fatigued conditions.

The follow-through phase commenced immediately after peak impact, involving progressive flexion of the elbow to approximately 30°–40° and anterior translation of the shoulder into slight flexion angles (~100°–110°). This deceleration phase was characterized by the dissipation of kinetic energy through controlled joint flexion, minimizing the risk of hyperextension injury. The reflective marker trajectories shifted inferiorly and posteriorly relative to the point of impact, consistent with biomechanical models of energy absorption following dynamic striking. Throughout the follow-through, the wrist remained stabilized to prevent loss of mechanical coupling between the fist and forearm.

All punch sequences were executed in a standardized environment, with fixed camera positioning ensuring orthogonal alignment to the sagittal plane. Measurement precision was reinforced through prior calibration, and all movement phases were verified by certified biomechanists to maintain consistency across trials.

Supplementary Text S2. Experimental Setup and Instrumentation Protocol

This supplement outlines the complete measurement protocol used for the assessment of upper-limb striking biomechanics in MMA athletes. The procedures were divided into three phases: (1) 2D motion capture during non-contact punching (shadow boxing), (2) 3D kinematic analysis under identical conditions, and (3) contact-based impact force assessment using a vertically mounted force plate. The step-by-step structure presented below allows for reproducibility and transparency in replication efforts.

Text-Based Experimental Setup Overview (Step-by-Step)

General Setup

- Participants: Elite MMA athletes (n = …), performing straight punches in controlled laboratory conditions.
- Environment: Flat, non-slip surface in a laboratory motion capture room with controlled lighting.

Phase 1 - 2D Kinematic Recording (Shadow Punching – No Contact)

Camera Setup

- 1 high-speed digital camera (120 Hz), mounted 1.2 m above ground level, at a distance of 2.0 m directly perpendicular to the sagittal plane of the athlete.
- Alignment was strictly orthogonal to the punching axis to minimize parallax error.

Calibration

- A 2D reference frame calibration using a standardized checkerboard or scale bar.

Marker Placement (2D)

- Reflective markers placed on the dominant arm: acromion, olecranon, ulnar styloid process.

Protocol

- Each athlete performed a series of shadow punches (no physical contact) to allow accurate motion capture without marker occlusion.

Data Recorded

- Angular displacement of shoulder and elbow joints in the sagittal plane.
- Frame-by-frame digitization using motion analysis software.

Phase 2 - 3D Kinematic Recording (Shadow Punching – No Contact)

System

- 8-camera Vicon 3D motion capture system (sampling at 200 Hz), positioned in a calibrated volume surrounding the athlete.

Marker Setup (3D)

- Full-body marker set or upper-limb cluster: key markers included acromion, lateral epicondyle, wrist styloid, third metacarpal head.

Calibration

- Volume calibrated using a static wand and dynamic wand procedure (standard Vicon calibration).

Reference Frame

- 3D joint angles computed in the global laboratory reference frame, projected to the sagittal plane for comparison with 2D.

Protocol

- Participants executed the same shadow punch trials as in the 2D phase.

Output

- Elbow and shoulder joint kinematics, analyzed in the sagittal plane for comparison with 2D data.

Phase 3: Impact Force Measurement Trials (Contact Punches – Force Plate)

Force Plate Setup

- Kistler 9287CA force plate (1000 Hz) vertically mounted on a padded wall, covered with 5 mm high-density foam to ensure safety.

Trial Structure

- Participants delivered maximal punches with the dominant arm into the padded force plate.
- These trials were conducted separately from motion capture sessions to prevent marker displacement.

Supplementary Instrumentation

- Noraxon Ultium accelerometer (1500 Hz) mounted on the striking glove to collect peak acceleration during both contact and non-contact trials.

Data Analysis

- Direct impact forces (Kistler) and indirect estimations (via velocity and impulse-momentum modeling) computed.
- Segmental mass estimated using Dempster's anthropometric tables.

Summary of Measurement Parameters

Parameter	Value/Method
2D camera distance/height	2.0 m / 1.2 m
2D sampling frequency	120 Hz
3D motion system	Vicon, 8 cameras, 200 Hz
Force plate	Kistler 9287CA, vertical, 1000 Hz
Accelerometer	Noraxon Ultium, 1500 Hz
Marker points	Acromion, Olecranon, Ulnar Styloid (+ others in 3D)
Analysis plane	Sagittal (global reference) for both 2D & 3D
